# Between here and there: comparing the worry about the pandemic between older Italian international migrants and natives in Switzerland

**DOI:** 10.1186/s40878-023-00331-6

**Published:** 2023-04-05

**Authors:** Sarah M. Ludwig-Dehm, Iuna Dones, Ruxandra Oana Ciobanu

**Affiliations:** 1 Faculty of Social Work (HETSL/HES-SO), University of Applied Sciences and Arts Western Switzerland, Ch. des Abeilles 14, 1010 Lausanne, Switzerland; 2 Swiss Centre of Expertise in Life Course Research, University of Geneva, Geneva, Switzerland; 3 Faculty of Social Work (HETS/HES-SO), University of Applied Sciences and Arts Western Switzerland, Geneva, Switzerland

**Keywords:** COVID-19, Migration, Worry, Transnationalism

## Abstract

Since the beginning of the pandemic in 2020, people have been worried about COVID-19. As one of the risk groups, persons aged 65 and older are especially vulnerable. Additionally, minorities and migrants are hit harder by the pandemic than natives. Using data from the TransAge survey, a study including over 3000 older persons (65+) living in Switzerland and Italy, we show that the levels of worry about the pandemic are significantly higher among Italian international migrants living in Switzerland than among Swiss natives. We are not able to fully explain the difference using sociodemographic variables, the COVID-19 situation at the time of the interview, and international migrants’ transnationalism behavior. Nevertheless, transnationalism explains a large part of the difference in worry between the two groups and our study sheds light on the importance of two specific transnational aspects, having Swiss nationality and voting behavior, for the prevention of elevated levels of worry of international migrants.

## Introduction

The COVID-19 pandemic has already badly affected everyone's life, through lockdowns, social isolation, contracting the coronavirus, and other ways. Often coming along with it is fear, anxiety, or worry about the pandemic. The worry can be felt for the society as a whole, for friends and family, or for oneself, to name just a few. Worrying about the pandemic can mean direct negative consequences for some people. While a certain level of worry is normal in these circumstances, studies show that excessive fear and worry are associated with negative health outcomes over time, like poor mental health and worse physical health (Garfin et al., 2020).

Not everyone is equally affected by the COVID-19 pandemic. Some societal groups, like older persons, are suffering more than other age groups in terms of worry and stress about the pandemic and their life satisfaction (Buyukkececi, 2021; Kulin et al., 2021). One of the reasons for this might be that because of their age they are considered a high-risk group, meaning that they have an increased risk of severe illness from COVID-19.

Research studies also show that minorities and migrants are hit harder than the native population in terms of health and financial consequences (Hu, 2020; Pew Research Center, 2020a) and risk perceptions (Soiné et al., 2021). These differences in the impact of the pandemic might aggravate already existing ethnic inequalities in society.

This paper investigates the worry about the COVID-19 pandemic of Swiss natives and Italian international migrants aged 65 years and older living in Switzerland. We define Swiss natives as individuals who were born and reside in Switzerland, and whose parents were also born in Switzerland. We define Italian international migrants as those born in Southern Italy and residing in Switzerland. The first part of the paper is answering the research question if international migrants living in Switzerland and Swiss natives worry to different degrees about the pandemic. In the second part, we are trying to answer the question about the causes for the differences in worry between these two groups. We examine three different explanations: composition effects, the COVID-19 situation at the time of the interview in the respondents’ region of origin, and international migrants’ transnationalism.

## Background

In 2020, the coronavirus spread rapidly around the globe, leading to a “public health emergency of international concern” (WHO, 2020). Up to writing this article (March 2023), more than 759 million infections worldwide have been confirmed and more than 6.8 million people have lost their lives due to the virus (WHO, 2023). In Italy, more than 188.000 people died, while in Switzerland more than 14.000 people lost their lives due to COVID-19 (Dong et al., 2020).

The rapid spread of the virus and the high infection and mortality rates worry people all around the globe (Sloan et al., 2021). In addition, countries’ responses to the pandemic, like school and border closures, travel restrictions, confinements, and a shutdown of large parts of the economy, have disrupted millions of lives and left people worried (Serrano-Alarcòn et al., 2021). They worry about the economy, about contracting the virus themselves, about exposure to the virus of family and friends, as well as finances, to name just a few (Buyukkececi, 2021; McCarthy, 2020; Pew Research Center, 2020b).

While a certain level of worry is a normal and healthy response to a situation like the COVID-19 pandemic, elevated levels can provoke numerous adverse consequences on a societal as well as an individual level. At the societal level, among individuals of all ages, heightened worry and anxiety can lead for example to panic shopping and xenophobia (Mertens et al., 2020). At the individual level, high levels of worry are found to be associated with various negative short-term and long-term outcomes for individuals’ physical health, psychological health, and psychosocial outcomes, including a poor general physical health (Garfin et al., 2018), increased pain (Garfin et al., 2018), reduced well-being and life satisfaction (Blix et al., 2021; Kivi et al., 2021; Özmen et al., 2021), raised stress (Blix et al., 2021; Lieberoth et al., 2021), depression (Garfin et al., 2018), other mental health problems (Mertens et al., 2020), and increased family conflict (Garfin et al., 2018). Several of these outcomes have already been found specifically due to the ongoing COVID-19 pandemic (e.g. Blix et al., 2021; Mertens et al., 2020), demonstrating the need for research and preventive measures in this area. These studies addressed individuals of all ages, with data collected specifically in Europe (Blix et al., 2021; Mertens et al., 2020) and North America (Mertens et al., 2020). To the best of our knowledge, there are no studies focusing on the impact of the COVID-19 pandemic on older European migrants. In this study, we focus on two of the most vulnerable societal groups, old people and migrants, to examine their levels of worry about the pandemic, in order to learn more about their determinants and preventive factors. We argue that age- and migration-related vulnerabilities combine in several ways.

First, older people are more vulnerable than other age groups. Studies focusing on worry and stress with the pandemic conducted on large world-wide samples (Buyukkececi, 2021), as well as individual countries (Kulin et al., 2021) show that older people worry more about the pandemic and are more stressed because of it. Because older age groups are one of the risk groups for COVID-19, meaning that they have an increased risk for severe illness, they might carry a higher psychosocial burden (Blix et al., 2021), which can lead to elevated stress levels and can have important consequences for older adults’ psychological and mental health (Tyler et al., 2021; Zaninotto et al., 2022).

Second, independently of the COVID-19 crisis, in-person contact with friends and family is an important contributor to older adults’ well-being (Cacioppo et al., 2010; Macdonald et al., 2021). This relationship between face-to-face contact and older adults’ well-being becomes especially important in the pandemic context, as older individuals were strongly recommended to isolate and minimize their face-to-face interactions. Although some older adults were able to maintain connections with friends and family through digital means, research on older adults in the United States (Hawkley et al., 2021) and across 27 European countries (Skałacka & Pajestka, 2021) has revealed that the use of remote communication means does not replace in-person contact in maintaining mental health. In fact, physical distancing during the pandemic has been shown to relate to higher levels of loneliness, depression, and anxiety in older adults in both Europe and the United States (Hawkley et al., 2021; Litwin & Levinsky, 2022; Tyler et al., 2021). Therefore, this decreased in-person contact may have contributed to older adults’ worry about the pandemic.

Third, age-related health problems require certain older adults to need formal or informal care (European FSO, 2020; Union, 2021), which were impacted by the pandemic and the regulations implemented to contain the virus (Tur-Sinai et al., 2021). Research has shown that in most European countries, older community-dwelling adults reported receiving more informal care by family, friends or neighbors since the outbreak of the pandemic. However, in some European countries like Italy, older individuals reported receiving less informal care (Tur-Sinai et al., 2021). Moreover, following the COVID-19 outbreak, older adults in most European countries reported having difficulties receiving formal home care from professionals that adequately met their needs (Tur-Sinai et al., 2021). The changes in care provisions engendered by the virus may have thereby influenced this population’s worry about the pandemic.

Fourth, the migration background adds a supplementary layer of vulnerability. Specifically in Switzerland, it was found that having Swiss nationality is associated with less stress during the COVID-19 pandemic and a higher life satisfaction compared to persons without Swiss nationality (Kuhn et al., 2021). Although the majority of migrants aged 65+ are retired and were not financially impacted by the pandemic through job loss, older Italian migrants in Switzerland have been found to have a lower socioeconomic status, lower education levels, and report themselves in worse health than Swiss natives (Bolzman & Vagni, 2018). Switzerland has an advanced health care system and mandatory health insurance, but it also has one of the highest health care costs in the world, making it less accessible to lower-income individuals (FSO, 2020; Tzogiou et al., 2021). Research shows that migrants in Switzerland are less likely than natives to visit a doctor, and this difference is largely explained by occupation and income (Tzogiou et al., 2021). Given Italian migrants’ lower socioeconomic status, the COVID-19 situation and the potential necessity of visiting doctors due to the pandemic may have consequently increased their worry about the health crisis.

Furthermore, Italian migrants’ lower education may have played a role in their worry with the pandemic. Lower education levels may influence health literacy (Rowlands, 2014; Ruedin et al., 2022), which is defined as “the cognitive and social skills which determine the motivation and ability of individuals to gain access, understand and use information in ways which promote and maintain good health” (Nutbeam, 2008, p. 2074). Although most migrants in Switzerland felt well informed about the pandemic, research has found that those with lower education levels had more difficulties in finding and understanding COVID-19 related health information (Ruedin et al., 2022). This may therefore have augmented their worry.

Based on these study results, we hypothesize that:

### **H1**

Italian international migrants living in Switzerland are more worried about the COVID-19 pandemic than Swiss natives.

We investigate three different explanations for the difference in worry about the pandemic between Italian international migrants and Swiss natives: (1) composition effects, (2) the COVID-19 situation in the region of origin as well as in the canton of residence, and (3) the transnationalism of international migrants.

Composition effects could explain the difference in worry between Swiss natives and Italian international migrants, as Italian migrants in Switzerland generally have a lower socioeconomic status, lower education levels, and are in worse health than Swiss natives (Bolzman & Vagni, 2018), and these sociodemographic charactertistics are found to be associated with varying levels of stress and worry (Kuhn et al., 2021; Mertens et al., 2020; Soiné et al., 2021). Studies find that persons having a higher salary experience in general less stress during the COVID-19 pandemic compared to those with a lower salary (Kuhn et al., 2021). Research shows that higher education is associated with lower stress levels during the pandemic (Kuhn et al., 2021), and that worse overall health and self-rated health are associated with more fears about COVID-19 (Mertens et al., 2020; Soiné et al., 2021). Other sociodemographic variables that could vary between groups and are found to be associated with differing stress and worry levels are age, gender, happiness, family situation, canton of residence in Switzerland, as well as religiosity. Being older is associated with more perceived stress and worry about the COVID-19 pandemic (Kuhn et al., 2021; Kulin et al., 2021). Although our dataset only consists of persons aged 65 and older, we assume that the worry about the pandemic is different between young–old and old–old individuals (Kuhn et al., 2021). Women are found to be on average more worried and stressed than men (Buyukkececi, 2021; Czymara et al., 2021; Kulin et al., 2021). Similar to self-rated health, happiness is associated with stress and worry: studies show that older adults’ well-being is associated with their level of worry, with lower well-being meaning a higher level of worry (Kivi et al., 2021). Having a partner or being married is found to be associated with lower levels of anxiety, worry, and stress regarding the pandemic (Buyukkececi, 2021). On the other hand, having children is found to increase worry, stress, and anxiety (Kerr et al., 2021; Westrupp et al., 2021). Religiosity might increase due to a crisis like the COVID-19 pandemic and can give comfort to individuals (Molteni et al., 2021) by reducing worry about the pandemic (Kulin et al., 2021). Finally, the respondents' canton of residence might account for possible regional differences that might have an effect on the worry about the pandemic, like the regional COVID-19 situation (Kuhn et al., 2021).

Although the two populations in this study might differ on these sociodemographic characteristics, they do not differ strongly on them, and we think that these differences cannot entirely explain the difference in worry between the two groups. Therefore, we hypothesize, that:

### **H2**

Composition effects explain only a small portion of the difference in worry between Italian international migrants and Swiss natives.

An already considerable amount of research studies has been published since the beginning of the pandemic linking the pandemic to negative health outcomes, lower well-being, and more worry and stress (see for example Blix et al., 2021; Garfin et al., 2020; Hu, 2020; Sloan et al., 2021). Yet the exact reasons for this link are still debated. While some research shows a link between the severity of the pandemic and health outcomes (Le & Nguyen, 2021), others argue that this link is mediated by policy responses (Serrano-Alarcòn et al., 2021).

Le and Nguyen (2021) argue that the pandemic per se affects individuals’ psychological well-being through various channels. For example, higher infection or hospitalization rates or higher mortality associated with COVID-19 directly lead to an increased fear about contracting the virus. The authors’ study links higher mortality rates to more dissatisfaction and higher levels of worry and anxiety. On the other hand, Serrano-Alarcòn and colleagues (2021) find in their quasi-natural experiment that individuals’ mental health is more affected by the containment policies as a response to the pandemic than by the severity of the pandemic itself.

However, concerning our sample, both of our populations, Italian international migrants and Swiss natives, are residing in Switzerland, meaning that the context of the pandemic is the same in terms of severity of the pandemic as well as in terms of policy responses. The difference between these two populations is the fact that one of them is linked through their migration to Italy. Italy is one of the countries that has been hit very early on in the pandemic and was hit the hardest during the beginning of 2020 (Remuzzi & Remuzzi, 2020). Additionally, research shows that one of the biggest fears in the context of COVID-19 is the health of others, like friends and family (Mertens et al., 2020). This fear even ranks above the worry about one's personal health.

Italian international migrants living in Switzerland, although having migrated decades ago and having aged in place, still have friends and family in Italy. We suspect that the severity of the COVID-19 situation explains all or almost all of the difference in worry between the two population groups. Therefore, we hypothesize, that:

### **H3**

The COVID-19 situation in the region of origin explains a large part of the difference in worry between Italian international migrants and Swiss natives.

Migrants are linked to various degrees and in various ways to their country of origin, often referred to as transnationalism (Glick Schiller et al., 1992; Levitt & Jaworsky, 2007; Portes et al., 1999). Transnationalism comprises many different feelings and activities, and is operationalized in countless ways, like sending remittances to the country of origin (Carling & Pettersen, 2014; Schunck, 2011), voting in the country of origin (Guarnizo et al., 2003), return intentions (Carling & Pettersen, 2014), and communication with friends and family in the country of origin (Järv et al., 2021), to name just a few.

Due to policy responses to the pandemic, like travel restrictions and border closures, transnational practices have likely changed since the onset of the pandemic (Nehring & Hu, 2021). Research is still missing on how migrants’ transnationalism affects their well-being during the pandemic. In line with the transnational social protection perspective (Faist et al., 2015; Levitt et al., 2017), it can be argued that transnationalism can serve as a social protection against problems and uncertainties posed by the COVID-19 pandemic (Cao & Sun, 2021). In contrast to this perspective, it can be argued that a link with the country of origin can also entail a ‘double exposure’ to information and news about the pandemic from two different contexts. Cao and Sun (2021) find in their research, for example, that watching news and being in contact with family and friends in China helped Chinese migrants in the U.S. being prepared for the outbreak through fostering their anxiety and risk perception. Additionally, having friends and family in Italy can increase the worry of international migrants who care for the health of those left behind (Mertens et al., 2020).

Switzerland and Italy experienced the pandemic in quite different manners. While Switzerland’s COVID-19 containment measures were relatively lax, Italy implemented more stringent containment measures, with a complete lockdown from March to May of 2020, and other restrictions thereafter (Bosa et al., 2022). During this time, older adults in Southern Italy reported decreased physical activity, worse eating habits, as well as worse dietary habits in comparison to pre-lockdown behaviors, and these may have meaningful health consequences in the long run (Gallè et al., 2021). Because of migrants’ ties to the home country and the implications that a strict lockdown may have had on individuals’ well-being, we suspect that Italian international migrants’ transnationalism explains all or almost all of the difference in worry between the two population groups. Therefore, we hypothesize that:

### **H4**

Italian international migrants’ transnationalism explains a large part of the difference in worry between Italian international migrants and Swiss natives.

## Data and methods

Data for this study comes from the Transnational Ageing project (“Transnational Ageing among Older Migrants and Natives: A Strategy to Overcome Vulnerability”), in which data was collected from four different populations in Switzerland and Italy: (a) from Swiss natives, born and residing in Switzerland, (b) from Italian international migrants, born in Southern Italy and residing in Switzerland, (c) from Italian internal migrants, born in Southern Italy and residing in Northern Italy, and (d) from Italian non-migrants, born and residing in Southern Italy. All respondents are aged 65 years and older. The data was collected between June and November 2020, during the COVID-19 pandemic, between the first and the second wave of infections. For the data collection in Switzerland, a sample from the Swiss Federal Statistical Office was used. The sample is stratified by age and gender. Stratification by canton was not possible, therefore, the six cantons in which almost 70% of the total Italian resident population resides were chosen: Zurich, Bern, Aargau, Vaud, Geneva, and Ticino. The respondents were contacted by letter and had the choice between an online and a paper questionnaire, each one available in German, Italian, and French. Our main models include the two populations living in Switzerland: Italian international migrants and Swiss natives. The final sample includes 716 Swiss natives and 513 Italian international migrants.

### Dependent variable

Our dependent variable in this study is worry about the pandemic. In general, worry is often measured using a comprehensive instrument, like the Penn State Worry Questionnaire (Meyer et al., 1990). But in time-limited contexts, worry can also be measured using just one item (Schroder et al., 2019). Additionally, because we are only looking at one specific aspect of worry, i.e. worry about the COVID-19 pandemic, in this study we measure it using one single survey item. Our measure is similar to the item capturing worry about the coronavirus in the Fear of the Coronavirus Questionnaire (Mertens et al., 2020) and the same measure is used in the study by Kulin and colleagues (2021). All respondents in our study were asked, “Please indicate on a scale from 0 to 10”, how much the COVID-19 pandemic has worried you. 0 means “not at all worried” and 10 means “very worried”. The coding of this variable is kept in the analysis, with higher values standing for more worry.

### Independent variables

The first set of independent variables is used to examine if composition effects can explain the difference in the worry about the pandemic between international migrants and natives. As explained above, we use several different variables that have been found to be associated with varying levels of stress and worry and on which the two populations might differ. These variables are the respondents’ age, gender, health, happiness, financial situation, education, family situation, canton of residence in Switzerland as well as their religiosity. Age is measured in years. To measure the respondents' health, we rely on a self-rated health measure asking them how they rate their general health on a 5-point Likert scale. The final variable distinguishes between good, average, and bad self-rated health. To measure happiness, respondents were asked to rate their general happiness on an 11-point Likert scale. To avoid too many missing values in our sample, we choose to measure the respondents' financial situation using a variable asking the respondents how easy or difficult it is for them to make ends meet. The final variable distinguishes between easy, average, and difficult. Education is measured as the respondents' highest obtained degree and is coded as low, average, and high. To consider the respondents' family situation, we include two variables indicating whether a respondent has a partner and children. Respondents' religiosity is taken into account by measuring whether or not they pray regularly (i.e. at least once per month). To control for possible language effects when testing our transnationalism hypothesis, we also include a variable indicating whether respondents speak Italian.

To examine our second possible explanation for differences in worry between international migrants and natives, we use a variable to take the COVID-19 situation in the international migrants' Italian region of origin into account. Politics as well as studies use different indicators of the pandemic to assess and judge the severity of the situation, like new daily or weekly positive cases (Breznau, 2021; Buyukkececi, 2021; Serrano-Alarcòn et al., 2021) or deaths (Buyukkececi, 2021; Le & Nguyen, 2021; Serrano-Alarcòn et al., 2021). In this study, we use the total COVID-19 related deaths that occurred since the start of the pandemic. Using the total deaths as an indicator for the severity of the COVID-19 situation instead of another measure has several advantages: first, it is a measurement that is easily understood by people. Second, it is often reported in the media and the news and, therefore, familiar to people (Le & Nguyen, 2021). Third, it gives a picture of the pandemic that is not only taking the present situation into account, but also the past development. And fourth, given that testing for COVID-19 infections was not widely available during the first months of the pandemic, using the total deaths is the best method for measuring the severity of the pandemic in our study (Le & Nguyen, 2021).

The data for this variable comes from official data from the Italian Department of Civil Protection and the Swiss Federal Office of Public Health and is published on a daily basis. Specifically, the variable indicates the total COVID-19 deaths in the respondent's region of birth per 100,000 citizens that occurred from the beginning of the pandemic until the respondent's interview. To avoid daily variations, we use the weekly average. For respondents who filled out paper questionnaires and sent them in, we assume that the time of responding to the survey happened in the same week in which the questionnaire was received.

With the use of the severity of the pandemic in the Italian migrants’ regions of birth, we assume that the respondents still have relations to Italy, and more specifically to their region of birth. The use of the region of birth is also due to the fact that the dataset does not contain information on relations to other regions in Italy besides the region of birth. Supporting our assumption, for more than two thirds of our final sample, we find a relation with their region of birth, like staying there for vacation, celebrating holidays related to their region of birth or remitting to someone who lives in their region of birth. Therefore, we assume that the region of birth is the best location available in the data to measure the severity of the pandemic. In contrast, for the group of Swiss natives, the canton of residence is used instead of the canton of birth, assuming that the COVID-19 situation in the canton in which someone lives is more important to them than in the canton of birth.

Our final set of independent variables measures transnationalism of international migrants in Switzerland. We use eleven different variables to form three types of transnationalism. The variables are often used to study transnational behavior of migrants and include several different dimensions of transnationalism, like return intentions (Carling & Pettersen, 2014), membership in clubs and organizations related to the country of origin (Snel et al., 2006), and remittances (Carling & Pettersen, 2014; Schunck, 2011). The variables are: (1) whether the respondents have close family living in Italy, meaning children, siblings, or parents, (2) whether they hold the Italian nationality, (3) whether they speak Italian, (4) whether they have stayed in Italy during the past 12 months, (5) whether they consider returning to Italy, (6) whether they want to be buried in Italy, (7) whether they celebrate holidays from Italy, (8) whether they have voted in the last Italian national election, (9) whether they are remitting to friends or family in Italy, (10) whether they watch TV, listen to the radio, or read the newspaper from Italy, and (11) whether they own a house in Italy.

All of the variables are coded in a binary way, differentiating between respondents who show the specific transnational behavior and respondents who do not show it. To capture the underlying structure of transnationalism among older Italian migrants in Switzerland, we employ latent class analysis (LCA). The advantage of this method is that the different classes do not have to be ordered along a continuum but can be interpreted as different types of transnationalism (Ayalon, 2016; Bacher & Vermunt, 2010; Ciobanu & Ludwig-Dehm, 2020). The different types can be described using the conditional probabilities of each included variable (Silverstein & Bengtson, 2002).

We employed the LCA without prior assumptions regarding the number of latent classes and chose the three-class solution. The three-class solution shows the lowest BIC and aBIC value. Additionally, the classes are interpretable and while the size of the groups is still manageable, it differentiates better than the two-class solution.^1^ All variables are summarized in Tables 1 and 2. The final sample size includes 1229 respondents, 513 international migrants and 716 Swiss natives.

**Table 1:** Descriptive statistics of categorical variables

Variable	International migrants		Swiss natives	
	N	%	N	%
Gender				
Female	217	42.30	304	42.46
Male	296	57.7	412	57.54
Health^a^				
Good	243	47.37	536	74.86
Average	229	44.64	154	21.51
Bad	41	7.99	26	3.63
Making ends meet^a^				
Easy	132	25.73	448	62.57
Average	227	44.25	232	32.40
Difficult	154	30.02	36	5.03
Education^a^				
Low	323	62.96	60	8.38
Medium	144	28.07	386	53.91
High	46	8.97	270	37.71
Having a partner				
Yes	403	78.56	563	78.63
No	110	21.44	153	21.37
Having a child^a^				
Yes	494	96.30	603	84.22
No	19	3.70	113	15.78
Praying^a^				
Yes	307	59.84	310	43.30
No	206	40.16	406	56.70
Canton of residence^a^				
Zurich	97	18.91	219	30.59
Bern	50	9.75	195	27.23
Geneva	62	12.09	43	6.01
Vaud	81	15.79	120	16.76
Ticino	174	33.92	45	6.28
Aargau	49	9.55	94	13.13
Italian language				
Yes	506	98.64	183	25.56
No	7	1.36	533	74.44
Transnationalism				
Class 1	259	50.49		
Class 2	31	6.04		
Class 3	223	43.47		

*Source*: TransAge Survey 2020

^a^Significant difference between international migrants and Swiss natives

**Table 2:** Descriptive statistics of continuous variables

Variable	International migrants				Swiss natives			
	Min	Max	∅	SD	Min	Max	∅	SD
Worry about COVID-19^a^	0	10	7.46	2.68	0	10	5.65	2.69
Age	65	96	74.32	6.2	65	96	74.89	6.62
Happiness^a^	0	10	7.66	1.84	0	10	8.14	1.51
COVID-19 deaths in region of birth^ab^	4.67	38.36	10.46	8.54	6.58	89.05	21.39	23.05

*Source*: TransAge Survey 2020

^a^Significant difference between international migrants and Swiss natives

^b^This variable indicates the COVID-19 deaths in the canton of residence for Swiss natives

### Methods

To answer our first research question, we rely on chi-square tests and t-tests as well as basic linear regressions to compare the means of worry between the different populations. To answer our second question, we compute several linear regressions. In a first model, we include only the variables to examine the possibility of a composition effect. In a second model, we additionally include our COVID-19 indicator. We keep the variables from the first model in this and all following models in which they serve as control variables. Finally, in a third model, we include our transnationalism measure along with the measure for Italian language. All models are computed using clustered standard errors to take the clustering between cantons into account.

## Results

**Fig. 1:**
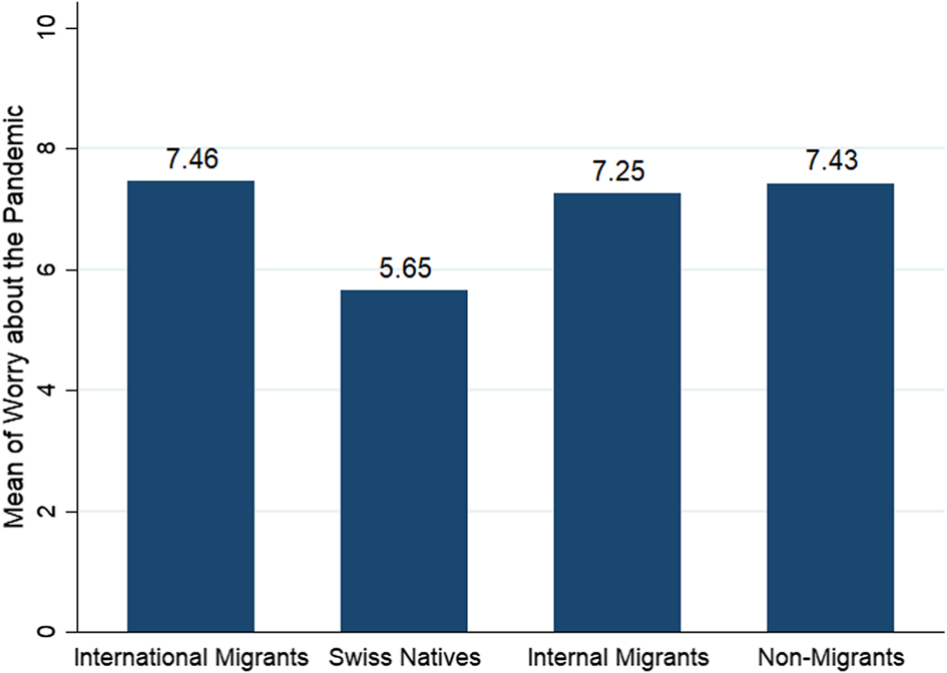
Comparison of mean of worry about the pandemic between survey populations

As a first step in understanding the worry about the pandemic of the different populations in our survey, we look at the worry about the pandemic for each group. Figure 1 reports the means of the worry for each population. Two observations are noteworthy from this figure. First, supporting our first hypothesis, Italian international migrants living in Switzerland are on average more worried about the pandemic than Swiss natives. The difference with 1.81 points is quite large (mean of 7.46 for international migrants and 5.65 for Swiss natives). Although both of these populations live in Switzerland and live through the COVID-19 pandemic in the same country, they seem to experience it very differently. And second, interestingly, the mean of worry for international migrants is very similar to the mean of the two Italy-based populations in the survey: internal migrants and non-migrants.

In the following, we will examine the three possible explanations discussed above for the difference in worry between international migrants and Swiss natives.

### Composition effects

As described above, the composition between migrants and natives could be one reason why we see a difference in worry about the pandemic. Therefore, we test whether certain variables, like gender, education, and financial means, can explain the worry difference. Tables 1 and 2 show the means of all variables separately for international migrants and Swiss natives. The variables for which the t-test or chi-square test shows a significant difference between the two populations are marked in the tables. Most of the variables differ between the two populations: international migrants are more likely than Swiss natives to report average or bad health; for a lot more international migrants than Swiss natives it is difficult to make ends meet; they are more likely to have a low educational level, to have children, and to pray regularly; international migrants in this sample are more likely to reside in the cantons of Geneva and Ticino, while Swiss natives are more likely to live in Zurich, Bern, and Aargau. Additionally, international migrants report lower happiness compared to Swiss natives.

To test whether the differences in these variables contribute to the difference in worry about the pandemic between the two populations, we compute two linear regressions that are reported in Table 3. In Model 1 in Table 3 no variables are included except for the main independent variable, the populations. In line with the first hypothesis and the results of Fig. 1, we see that the difference in worry about the pandemic between Swiss natives and international migrants is 1.806 points. This difference is statistically significant.

**Table 3:** Linear regressions to test composition effects on worry about the pandemic

	Model 1—empty	Model 2—composition
Swiss natives (*ref.: international migrants*)	− 1.811** (0.332)	− 1.366** (0.287)
Age		− 0.015 (0.011)
Gender		
Female (*ref.: male*)		0.389* (0.124)
Health		
Average (*ref.: good*)		0.328 (0.184)
Bad		0.498 (0.267)
Making ends meet		
Average (*ref.: difficult*)		− 0.174 (0.455)
Easy		− 0.666 (0.419)
Having a partner		0.373 (0.226)
Having children		0.418 (0.364)
Education		
Medium (*ref.: low*)		− 0.053 (0.082)
High		− 0.128 (0.211)
Happiness		0.051 (0.081)
Praying regularly		0.124 (0.151)
Residence canton		
Zurich (*ref.: Bern*)		− 0.474*** (0.044)
Aargau		− 0.732*** (0.044)
Vaud		− 0.77*** (0.072)
Geneva		− 0.953*** (0.097)
Ticino		− 0.047 (0.177)
Constant	7.458*** (0.193)	7.734*** (0.867)
N	1229	1229
R^2^	0.0997	0.1379

*Source*: TransAge Survey 2020, own calculations

Standard errors in parentheses

*p < 0.05, **p < 0.01, ***p < 0.001

Model 2 includes all the variables mentioned above. The results show that, in line with the literature (Czymara et al., 2021), women are on average more worried about the pandemic than men. Additional, we find that the worry differs between the different cantons, with respondents living in Bern and Ticino being the most worried. Because the cantons can be a proxy for numerous regional differences, their significant effects cannot be definitely interpreted. Characteristics that have been previously associated with greater stress and fear about the pandemic like health, education, financial situation, and happiness do not show significant coefficients in the model.^2^ Comparing the coefficients for the populations, we see that it reduces in Model 2 to 1.37 points. Although we see a small reduction of the difference in worry about the pandemic between Swiss natives and international migrants, the difference is still rather high. We also see that the dependent variable’s variance that can be explained by the covariates included in the model increases slightly from Model 1 to Model 2 (from 0.0997 to 0.1379). Therefore, we find support for our second hypothesis that composition effects only explain a small portion of the difference in worry between the two groups.

### COVID-19 situation

To test our second explanation, we compute a third linear regression in which we also control for the COVID-19 situation in the respondents’ region of birth. Because we do not have information on the Swiss natives’ canton of birth, for this group we will use instead the canton of residence.^3^ The results are presented in Table 4.

**Table 4:** Linear regression to test COVID-19 indicator on worry about the pandemic

	Model 3—COVID-19
Swiss natives (*ref.: international migrants*)	− 1.197** (0.214)
COVID-19	− 0.008 (0.006)
Age	− 0.014 (0.011)
Gender	
Female (*ref.: male*)	0.393* (0.123)
Health	
Average (*ref.: good*)	0.339 (0.184)
Bad	0.523 (0.259)
Making ends meet	
Average (*ref.: difficult*)	− 0.174 (0.454)
Easy	− 0.674 (0.417)
Having a partner	0.366 (0.224)
Having children	0.417 (0.367)
Education	
Medium (*ref.: low*)	− 0.054 (0.08)
High	− 0.142 (0.213)
Happiness	0.053 (0.079)
Praying regularly	0.125 (0.148)
Residence canton	
Zurich (*ref.: Bern*)	− 0.46*** (0.037)
Aargau	− 0.724*** (0.037)
Vaud	− 0.608** (0.138)
Geneva	− 0.735** (0.137)
Ticino	0.184 (0.182)
Constant	7.588*** (0.831)
N	1229
R^2^	0.1394

*Source*: TransAge Survey 2020, own calculations

Standard errors in parentheses

*p < 0.05, **p < 0.01, ***p < 0.001

Model 3 shows that although the coefficient of the COVID-19 indicator is not significant, the coefficient of the group, i.e. for Swiss natives, decreases compared to Model 2. However, we only see a slight increase in R^2^, the variance of worry of the pandemic that can be explained by the model. Therefore, we can conclude that we do not find support for our third hypothesis, meaning that the COVID-19 situation in the respondents’ region of birth does not help explain their worry about the pandemic.

### Transnationalism

**Fig. 2:**
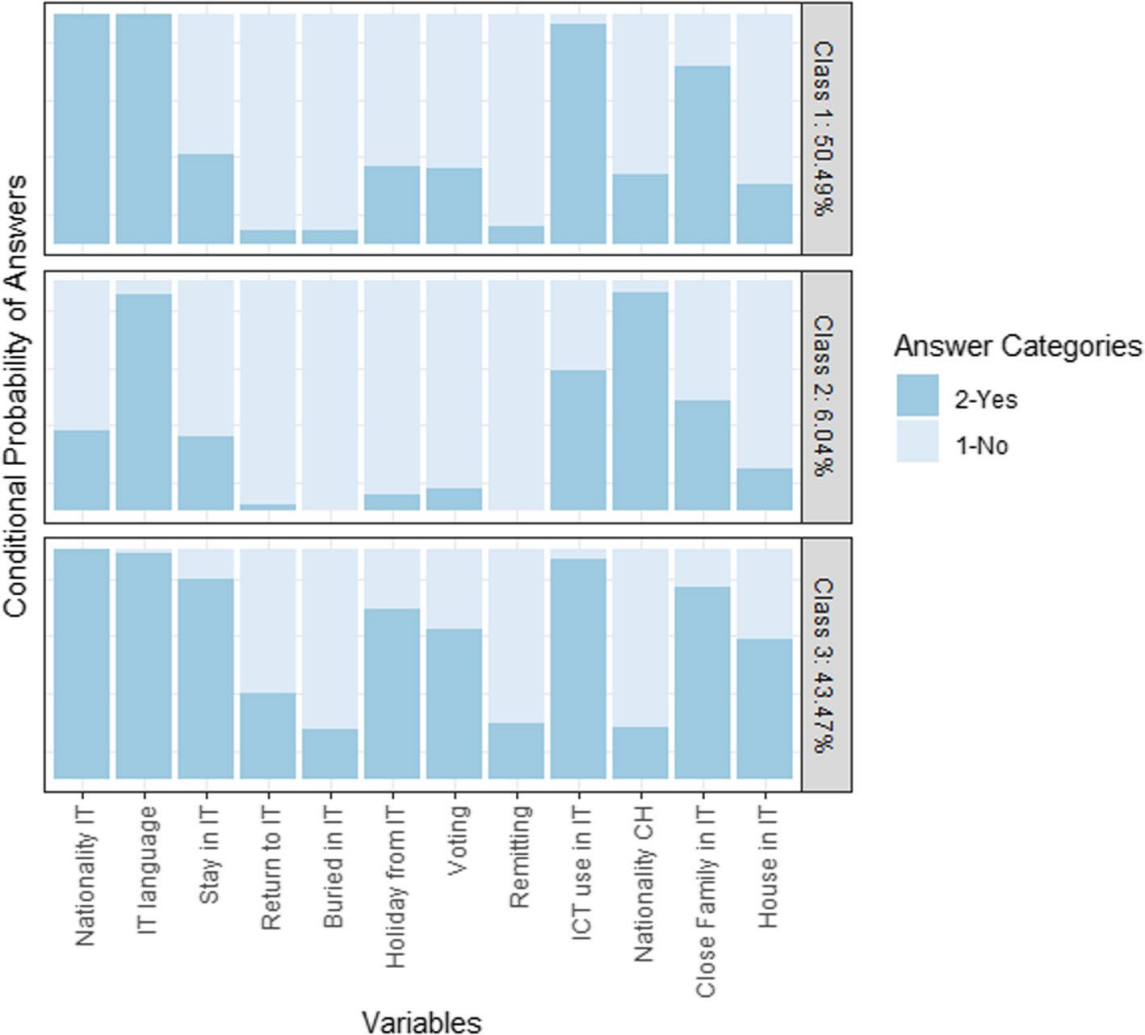
Conditional probabilities for LCA model with three latent classes

To test our third explanation, we turn to transnationalism of the international migrants in Switzerland. Transnationalism is measured using three latent classes that represent three different types of transnationalism. The three types are interpreted based on the conditional probabilities associated with the binary variables within each latent class, as shown in Fig. 2.

Class 3 of the LCA includes around 44% of the international migrants in our sample. They are the *Active Transnational*. The probabilities of showing transnational behavior are the highest across all eleven variables. Class 2, the *Detached*, include around 6% of the international migrants. They show stark differences in their transnational behavior compared to the *Active Transnational*, except for the Italian language usage. Importantly, only around one third of the *Detached*, still hold Italian nationality. In addition, almost all of the *Detached* international migrants hold Swiss nationality. Lastly, class 1 includes around 50% of the international migrants, who we call the *Passive Transnational*. While having relatively high probabilities of showing transnational behavior that can be done at a distance, like having the Italian nationality, speaking Italian, using ICT in Italian, or having close family in Italy, they are less likely than the *Active Transnational* to show other types of transnational behavior, like staying in Italy, wanting to return to Italy, and wanting to be buried in Italy.

**Fig. 3:**
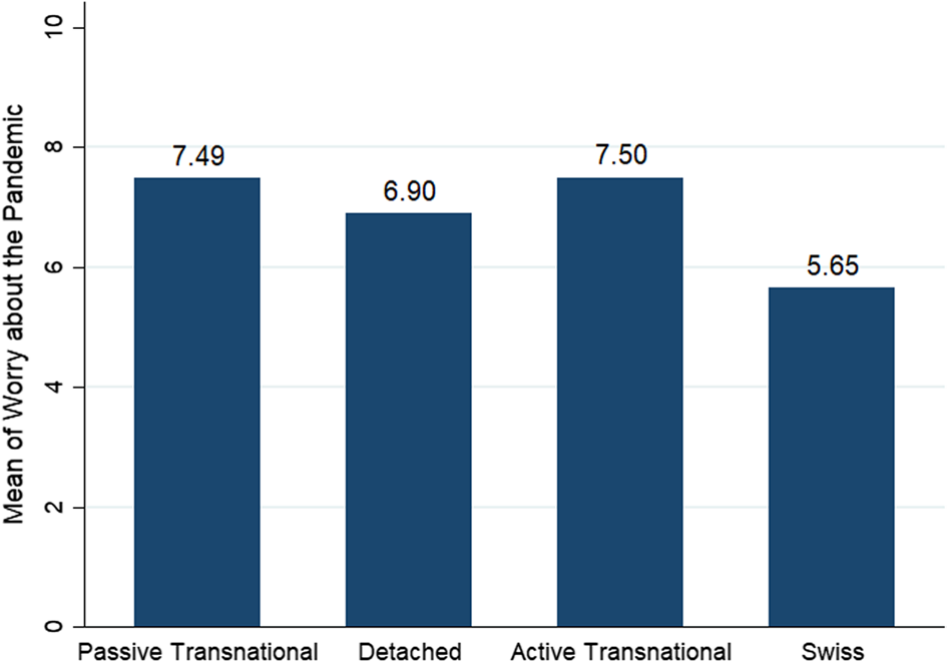
Comparison of mean of worry about the pandemic between transnationalism classes

To be able to use this categorical measure in our models, we add another category, which includes only Swiss natives. Figure 3 shows the mean of worry about the pandemic between the transnationalism classes and Swiss natives. The mean of worry is very similar for the *Active Transnational* and the *Passive Transnational.* Although the mean is a bit lower for the *Detached*, a t-test shows that the difference in the worry about the pandemic between the *Detached* and the Swiss natives is significant. The difference between the *Detached* and the *Active Transnational* as well as the *Passive Transnational* is not significant.

Model 4 in Table 5 shows a linear regression model including the transnationalism classes. To control for possible effects of Italian language use, in this model we also include the Italian language variable. The coefficients of the control variables do not change from Model 2 to Model 4. Looking at the coefficients for the different transnationalism types, we see important differences between them. Compared to Swiss natives, the *Active Transnational* and the *Passive Transnational* report a worry about the pandemic that is significantly higher (around 1.3 and 1.4 points on the worry scale). However, when looking at the third type of transnationalism, the *Detached*, we see that respondents from this type report a worry of the pandemic that is not significantly different from the one reported by Swiss natives.

**Table 5:** Linear regression to test transnationalism on worry about the pandemic

	Model 4—transnationalism
Transnationalism	
Active transnational (*ref.: Swiss natives*)	1.418** (0.231)
Detached	0.521 (0.219)
Passive transnational	1.313* (0.233)
Age	− 0.015 (0.011)
Gender	
Female (*ref.: male*)	0.407* (0.097)
Health	
Average (*ref.: good*)	0.345 (0.183)
Bad	0.513 (0.269)
Making ends meet	
Average (*ref.: difficult*)	− 0.179 (0.459)
Easy	− 0.666 (0.421)
Having a partner	0.367 (0.224)
Having children	0.413 (0.361)
Education	
Medium (*ref.: low*)	− 0.01 (0.078)
High	− 0.102 (0.233)
Happiness	0.057 (0.08)
Praying regularly	0.11 (0.158)
Speaking Italian	0.105 (0.359)
Residence canton	
Zurich (*ref.: Bern*)	− 0.499*** (0.053)
Aargau	− 0.740*** (0.043)
Vaud	− 0.78*** (0.075)
Geneva	− 0.996*** (0.095)
Ticino	− 0.05 (0.264)
Constant	6.254** (0.83)
N	1 229
R^2^	0.1402

*Source*: TransAge Survey 2020, own calculations

Standard errors in parentheses

*p < 0.05, **p < 0.01, ***p < 0.001

This result supports our third hypothesis that transnationalism explains a large part of the difference in worry about the pandemic between Italian international migrants and Swiss natives: Italian international migrants living in Switzerland who are detached from their country of origin are as worried about the pandemic as Swiss natives living in Switzerland. Furthermore, Italian international migrants who are more attached to Italy (i.e. the *Passive Transnational* and the *Active Transnational*) are significantly more worried about the pandemic than their Swiss native counterparts.

While supporting our hypothesis, this result does not explain what it is exactly about transnationalism that reduces or heightens the respondents' worry. Consequently, to shed more light on this issue, we will take a closer look at the different aspects of transnationalism and their relationship with the worry about the pandemic.

### Post hoc analyses

To examine in more detail the specific characteristics of transnationalism that might be responsible for diverse levels of worry about the pandemic, we compute additional models that are limited to the Italian international migrant population to explain their levels of worry about the pandemic. The additional analyses show that two variables measuring different aspects of transnationalism are standing out. These are the variable indicating if a respondent has voted in the last national elections in Italy and the variable indicating whether or not a respondent holds Swiss nationality. Respondents who have voted in the last national elections in Italy report a significantly higher worry about the pandemic compared to respondents who have not voted in the last Italian national elections. Regarding Swiss nationality, respondents who hold Swiss nationality report a significantly lower worry about the pandemic than respondents who do not hold Swiss nationality.

To test how much of the difference in worry about the pandemic between Swiss natives and Italian international migrants can be explained by only these two variables, we compute another model including both of these populations (see Model 5 in Table 6). For this model, a new variable is constructed that differentiates between (a) international migrants who have not voted in the last national elections in Italy *and* who hold Swiss nationality (“CH and not voted”, N = 95), (b) international migrants who have not voted in the last national election in Italy *or* who hold Swiss nationality (“CH or not voted”, N = 252), this group includes respondents who hold Swiss nationality and have voted and respondents who do not hold Swiss nationality and have not voted, (c) international migrants who have voted in the last national election in Italy *and* who do not hold Swiss nationality (“no CH and voted”, N = 166), and (d) Swiss natives (“Swiss”, N = 716).

**Table 6:** Additional analyses: linear regression to test voting and Swiss nationality on worry about the pandemic

	Model 5—additional analyses
Swiss nationality and voting	
CH *and* not voted (*ref.: Swiss*)	0.605* (0.191)
CH *or* not voted	1.264** (0.279)
No CH and voted	1.852** (0.257)
Age	− 0.017 (0.011)
Gender	
Female (*ref.: male*)	0.429* (0.114)
Health	
Average (*ref.: good*)	0.331 (0.179)
Bad	0.53 (0.259)
Making ends meet	
Average (*ref.: difficult*)	− 0.164 (0.42)
Easy	− 0.61 (0.371)
Having a partner	0.385 (0.218)
Having children	0.43 (0.369)
Education	
Medium (*ref.: low*)	0.017 (0.088)
High	− 0.072 (0.209)
Happiness	0.058 (0.081)
Praying regularly	0.111 (0.153)
Speaking Italian	0.09 (0.355)
Residence canton	
Zurich (*ref.: Bern*)	− 0.48*** (0.056)
Aargau	− 0.764*** (0.054)
Vaud	− 0.765*** (0.074)
Geneva	− 0.962*** (0.11)
Ticino	0.029 (0.24)
Constant	6.345** (0.812)
N	1229
R^2^	0.147

*Source*: TransAge Survey 2020, own calculations

Standard errors in parentheses

*p < 0.05, **p < 0.01, ***p < 0.001

Model 5 in Table 6 shows that the two variables, Swiss nationality and voting, are important for the worry about the pandemic. Although, even in this model, all of the three groups of Italian international migrants report a worry about the pandemic that is significantly higher than that of Swiss natives, we see stark differences between the three international migrant groups. Italian international migrants who have Swiss nationality and who have not voted in the last Italian national elections report on average a worry that is only 0.61 points higher than that of Swiss natives. Italian international migrants who have only either Swiss nationality or have not voted in the last national elections in Italy report on average a worry that is 1.26 points higher than that of Swiss natives. And finally, Italian international migrants who do not have Swiss nationality and who have voted in the last elections report on average a worry that is 1.85 points higher than that of Swiss natives. Additional analyses show that the differences between the three Italian groups are statistically significant.

These results are in line with our overall transnationalism results: Italian international migrants who are more transnational tend to be more worried about the pandemic. The additional analyses point to two important variables in this relationship, voting in the Italian national elections and holding Swiss nationality. But why are Italian international migrants living in Switzerland more worried about the pandemic if they have voted in the last Italian national elections and if they do not have Swiss nationality? We cannot fully answer this question with the data we have available. However, considering the meaning of these two characteristics of transnationalism for Italian migrants in Switzerland, we have some assumptions.

Persons voting in national elections are more interested in and more informed about current politics than non-voters (Delli Carpini & Keeter, 1996). Studies have also found that exposure to media (e.g. reading the newspaper, watching the news, or listening to the radio) is positively related to several forms of political participation, like voting (Corrigall-Brown & Wilkes, 2014; Smets & van Ham, 2013). Although research has paid little attention to external voting, especially to voting of emigrants of higher living standard countries (Peltoniemi, 2018), this should also be true for emigrants. As Mügge and colleagues (2021) find for Turks living in the Netherlands, their participation in elections in their country of origin is closely related to the availability of compatriot media. Other studies find similar relationships: Romanian and Polish migrants residing in Norway and Spain, express the importance of exposure to media—either through official media channels or through social media—for their political participation (Szulecki et al., 2021). Similarly, Peltoniemi (2018) finds in her study, that Finnish emigrants living abroad are more likely to vote in homeland elections if they are more interested in homeland politics.

This means that Italian migrants in our survey who report having voted in the last Italian national elections might be more likely to be interested in Italian national politics and more likely to be informed about day-to-day political news than Italian migrants who have not voted. This higher level of information of Italian migrant voters implies a detailed knowledge and awareness of the serious situation Italy was in during the first months of the COVID-19 pandemic in 2020. Additionally, other research points to the fact that migrant voters have a stronger identification with societal groups in the migrants' origin country and a closer relationship with the origin country in general (Lafleur & Sánchez-Domínguez, 2014). More information about and exposure to Italy's severe situation coupled with a strong identification and a close relationship with Italy and Italians, suggests that the Italian migrant voters are more worried about the pandemic for Italians in Italy. Therefore, the measure of having voted could be a proxy representing knowledge about the COVID-19 pandemic in Italy and the worry about the COVID-19 pandemic for Italians in Italy in general.^4^ This altruistic worry (worry felt for others) (Sloan et al., 2021) for Italians in Italy could be less present in migrant non-voters due to less exposure to Italian news coverage.

Contrary to this altruistic worry, holding Swiss nationality might be a proxy for a more personal worry (Sloan et al., 2021); a worry, respondents feel for themselves. Having the destination country’s nationality can be associated with a feeling of belonging to and an identification with the destination country (Kolbe & Crepaz, 2016; Schlenker, 2015). It also displays a form of detachment from Italy (Schlenker, 2015). In addition to this, especially holding citizenship from a country where the barriers to obtaining full citizenship status are comparably high (Koopmans, 2010), can add a layer of security for migrants. Having Swiss nationality might give migrants a secure feeling of being treated without being discriminated against in the Swiss health care system, something especially important during a pandemic. Although everyone in Switzerland has mandatory health insurance, out-of-pocket payments are exceptionally high and big health care inequalities exist between migrants and non-migrants (Tzogiou et al., 2021): non-migrants are more likely to visit a doctor and less likely to visit the emergency department (Tzogiou et al., 2021). In Switzerland, migrants are more likely than natives to lack information about the health care system and show lower participation in sickness-prevention activities (Spang & Zuppinger, 2010). First generation migrants are also more likely to not have a general practitioner, who acts as a gatekeeper to specialists and treatment in hospitals (Tzogiou et al., 2021). These studies point to barriers that are existent in the Swiss health care system preventing migrants from getting full health care (Tzogiou et al., 2021). Therefore, having Swiss nationality might give migrants the peace of mind to worry less about appropriate health care after a possible infection with COVID-19.

## Discussion and conclusion

In 2020, the coronavirus spread rapidly around the globe, having people worried due to the high infection and mortality rates (Sloan et al., 2021). While a certain level of worry is a normal and a healthy response to a pandemic, elevated levels can cause numerous adverse health consequences, like reduced well-being and life satisfaction (Blix et al., 2021; Kivi et al., 2021; Özmen et al., 2021), raised stress (Blix et al., 2021; Lieberoth et al., 2021), or depression (Garfin et al., 2018). The worry about the pandemic should, therefore, be treated as a public health concern.

In this paper, we investigated the worry about the COVID-19 pandemic of Swiss natives and Italian international migrants aged 65 years and older living in Switzerland. We found that although both of these groups live through the pandemic in the same country, Italian international migrants are significantly more worried about the pandemic than Swiss natives. We examined three different explanations: composition effects using sociodemographic variables, the COVID-19 situation at the time of the interview in the respondents’ region of origin, and international migrants' transnationalism.

Using these three different explanations, we are not able to fully explain the difference in worry between Swiss natives and Italian international migrants. However, we find that Italian international migrants who are detached from Italy have a level of worry about the pandemic that is not significantly different from the level of worry of Swiss natives. Additional analyses suggest that two variables are of importance in this context: whether the migrants hold Swiss nationality and whether they voted in the last national election in Italy. Reporting more detachment from Italy (i.e. holding Swiss nationality and not having voted) is associated with a significantly lower worry about the pandemic.

Holding Swiss nationality and having voted in the last Italian election could be proxies for two different types of worry. Having voted in the last Italian election could stand for an altruistic worry for family and friends living in Italy or for Italians in general. Migrants who voted in the last Italian election are likely more involved in Italian politics, following the news more regularly and therefore, are more informed and exposed to the severe situation Italy was in during the first months of the pandemic in 2020. Additionally, migrant voters are more likely to identify and have a close relationship with Italians (Lafleur & Sánchez-Domínguez, 2014). Holding Swiss nationality could stand for a personal worry or a lack thereof. Having Swiss nationality might give international migrants peace of mind regarding obtaining health care and other social services during the pandemic in Switzerland. Not having Swiss nationality might leave migrants feeling like second class citizens in an exceptionally expensive health care system with inequalities between migrants and non-migrants.

Our study does not come without limitations. First, our question on worry about the pandemic only captures overall worry without providing us with more precise information on the type of worry. Second, while our study shows the importance of two specific aspects of transnationalism for the worry of international migrants, the cross-sectional study design does not allow us to make causal claims. Future studies should explore this topic using longitudinal data. Third, our sample only includes a very specific group of international migrants living in Switzerland: Italian international migrants aged 65 years and older who are born in Southern Italy. Although Italians are the biggest foreign national group in Switzerland, this makes it difficult to generalize our results to the entire migrant population in Switzerland.

Another important aspect that has to be discussed in the context of limitations is the influence of the cultural and physical proximity to Italy. Swiss natives living in the Italian-speaking parts of Switzerland, might be more exposed to news about Italy and in 2020 to news about the COVID-19 situation in Italy. Although, in our analysis we controlled for Italian language use of Swiss natives, the sample in the canton of Ticino, which borders Italy and in which Italian is spoken, is too small to conduct separate analyses between the two groups of interest. Future studies should test whether transnationalism might still be important for levels of worry about the pandemic when cultural and physical proximity are controlled for.

Future research should investigate the worry about the pandemic with more precise measures of the type of worry. Additionally, we are calling for research studies exploring the exact mechanisms that link voting in the home country and holding the destination country's nationality and worry during a crisis like the COVID-19 pandemic. This research could inform policy makers of how to create environments that are inducing less worry, fear, and stress in migrants to reduce the emergence of adverse health outcomes.

## Data Availability

The Transnational Ageing dataset analyzed in this study is not yet available publicly. For now, it is available from Ruxandra Oana Ciobanu (Oana.Ciobanu@hetsl.ch) on reasonable request.

## Abbreviations

FSO: Federal Statistical Office
LCA: Latent class analysis
WHO: World Health Organization

## Footnotes

1 We computed a series of models, ranging from two latent classes to six latent classes that were compared using the BIC, aBIC, cAIC and the likelihood ratio. Our measures show that the two- and the three-class solution show the best fit to the data.

2 To acknowledge a possible reversed relationship of happiness and subjective health with worry about the pandemic, we also computed all models without these two variables. The results are nearly identical, and their interpretation stays the same.

3 We also computed a linear regression using the canton of residence for the whole sample (Swiss natives and international migrants). The results show that the coefficient for the COVID-19 indicator is not significant, the coefficient of the population (i.e. Swiss natives) does not change in size compared to Model 2 in Table 3, and the R^2^ does not increase compared to Model 2 in Table 3.

4 The knowledge and worry about the COVID-19 situation in Italy as a whole could be more important for the worry of the pandemic than the COVID-19 situation in the respondents’ regions of birth, explaining why we could not find a significant relationship between these two variables in Model 3.
